# An unusual case of an 0.018-inch (0.457 mm) guidewire fracture during primary percutaneous nephrostomy for percutaneous nephrolithotomy treated without removal and followed up for 5 years: A case report and literature review

**DOI:** 10.1097/MD.0000000000039281

**Published:** 2024-08-09

**Authors:** Seunghoon Oh, Bum Sang Cho, Yook Kim, Jisun Lee, Kyung Sik Yi, Sang-Cheol Lee

**Affiliations:** a Department of Radiology, Chungbuk National University Hospital, Cheongju, South Korea; b Department of Radiology, College of Medicine, Chungbuk National University, Cheongju, South Korea; c Department of Urology, College of Medicine, Chungbuk National University, Cheongju, South Korea; d Department of Urology, Chungbuk National University Hospital, Cheongju, South Korea.

**Keywords:** calyx, guidewire fracture, kidney, percutaneous nephrolithotomy, percutaneous nephrostomy

## Abstract

**Introduction:**

Although rare, guidewire fractures can occur during interventional procedures. In most cases, the fractured guidewire segment can be removed.

**Patient concerns:**

We report the case of a 54-year-old woman who experienced a guidewire fracture during percutaneous nephrostomy (PCN) for percutaneous nephrolithotomy to remove renal stones.

**Diagnosis:**

Nephrolithiasis.

**Interventions:**

PCN and percutaneous nephrolithotomy.

**Outcomes:**

In this case, the remaining segment could not be removed and caused inflammation and infection. However, her symptoms improved with inpatient treatment. Therefore, she was discharged from the hospital and followed up for 5 years.

**Conclusion:**

When performing PCN to remove renal stones, the possibility of a guidewire fracture must be considered. If resistance or scraping is felt while handling the guidewire, then it should be replaced.

## 1. Introduction

During interventional procedures, 0.018-inch (0.457 mm) guidewires are commonly used when navigating to the target lesion. Guidewire fractures during such procedures are rare complications, with fewer than 40 cases reported.^[1–4]^ In most cases, fractured guidewire segments that remain inside patients can be removed by interventions such as surgery; however, in rare instances, the retained segments cannot be removed.^[2–4]^ To the best of our knowledge, cases involving guidewire fractures during percutaneous nephrostomy (PCN) for percutaneous nephrolithotomy (PNL) to remove renal stones, especially staghorn stones, and failure to remove the retained fractured segment have not been reported. However, we encountered such a case.

## 2. Case report

**Figure 1.**
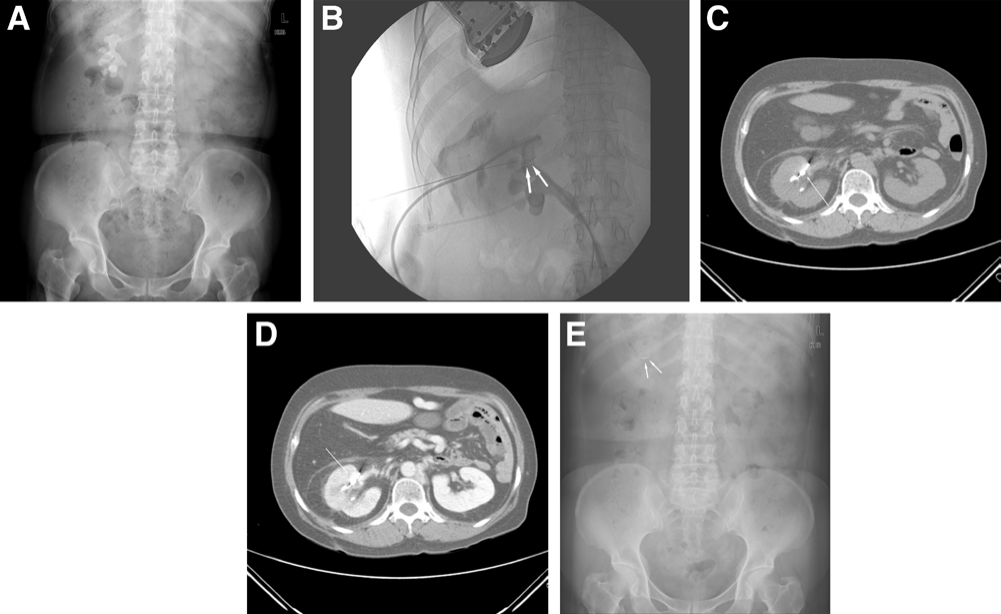
A 54-year-old female patient with a staghorn stone in the right kidney underwent percutaneous nephrostomy (PCN) for percutaneous nephrolithotomy (PNL). (a) The initial kidney, ureter, and bladder (KUB) study image demonstrated a large staghorn stone in the right renal pelvocalyx. (b) A fluoroscopic image obtained during PCN for PNL shows the fractured segment of the guidewire (arrow) at the upper renal calyx of the right kidney. A pigtail catheter was inserted at the upper renal calyx and another yellow sheath was inserted at the lower renal calyx. Leaked contrast media is observed in the right perirenal space. (c) A precontrast computed tomography (CT) image obtained 6 months postoperatively shows the retained fractured guidewire segment (arrow). The fractured fragment appears between the renal parenchyma and renal pelvis. (d) A contrast-enhanced CT image obtained 6 months postoperatively shows increased perirenal fat stranding and decreased enhancement of the right renal parenchyma indicating an inflammatory condition. The fractured fragment (arrow) appears between the renal parenchyma and renal pelvis. (e) The KUB study image obtained 5 years postoperatively shows the retained fractured guidewire segment (arrow).

A 54-year-old female patient was admitted to our institution for treatment of a right renal stone. She reported bilateral flank pain and had previously undergone extracorporeal shock wave lithotripsy for a left ureter stone at another hospital several days previously. A physical examination, urinalysis, and blood test performed at admission showed no abnormal findings. The findings of the kidney, ureter, and bladder (KUB) radiograph performed at our hospital and computed tomography (CT) performed at another hospital revealed a staghorn stone filling the right renal pelvis and calyx (Fig. 1a).

We performed PCN for PNL. After puncturing the posterior upper calyx with a 21-gauge biopsy needle (SHS-2115C; M.I. Tech, Seoul, Korea) under ultrasound guidance, contrast medium was injected to confirm that the needle tip was placed in the calyx. Subsequently, when the 0.018-inch (0.457 mm) guidewire (Merit Medical, West Jordan, UT) was inserted in the calyx, severe resistance was caused by the stone. During several attempts to move the guidewire, the needle tip was obstructed and could not be advanced or retracted. After several rounds of simple maneuvers (traction, pulling, and pushing), the guidewire could not be pulled out. We felt the guidewire tearing apart when we attempted to pull the puncture needle and guidewire together, and only the proximal portion of guidewire was removed. Fluoroscopic imaging confirmed that the fractured segment, which was the covered portion of the distal end of the guidewire, remained inside the kidney (Fig. 1b). After puncturing the same calyx again, we attempted removal using alligator forceps and a snare. However, because we could not access the fractured segment, PCN was performed at 2 sites on the upper calyx and lower calyx to complete the procedure (Fig. 1b). Despite our removal attempts using rigid and flexible ureteroscopes in the operating room, the fractured segment of the guidewire could not be found. Therefore, only the right kidney stone was removed, and the patient was discharged.

Six months later, the patient presented to our emergency department because of fever and nausea. The urinalysis and blood test findings indicated infection (complete blood count results: white blood cell count, 11.5 × 10^3^/µL; hemoglobin level, 11.1 g/dL; platelet count, 198 × 10^3^/µL; and C-reactive protein level, 17.15 mg/dL; urinary albumin: white blood cell count 3+, blood 3+). Abdominopelvic CT showed a fractured segment on the parenchyma of the upper pole of the right kidney and surrounding perirenal fat space as well as increased perirenal fat stranding and decreased enhancement of the right renal parenchyma. Based on these findings, we suspected pyelonephritis (Fig. 1c and d). Subsequently, the patient was hospitalized and treated with antibiotics. Her symptoms improved, and she was discharged 5 days later. After discharge, we monitored her condition during outpatient follow-up visits. During 5 years of outpatient follow-up, the retained wire was visible during the KUB radiograph; however, the patient did not report any specific complications (Fig. 1e).

## 3. Discussion

The basic structure of a guidewire consists of an inner core, a proximal end made of steel, and a short distal end covered by nitinol. The nitinol is in the form of a spiral coil that wraps around the core.^[5]^ In our case, the covered portion of the distal end remained inside the patient.

Guidewire fractures are rare complications that can occur during an interventional procedure. Previously, only fractures that have occurred during coronary artery interventions (0.1%–0.2% occurrence rate) have been reported.^[6]^ These fractures may occur when the tip catches on the valve, calcified portion, or intimal layer, when severe vessel tortuosity and calcification exist, or when a long stent causes stent entrapment.^[7]^ Additionally, guidewire fractures may be caused by severe coronary spasms or manufacturing defects.^[8]^ Guidewire (0018–0.035 inches (0.457–0.889 mm)) fractures that occurred during endoscopic retrograde cholangiopancreatography have been reported as well.^[9]^ According to a previous study,^[10]^ a snare loop (32.1%), double-wire or triple-wire technique (10.7%), and stenting against the vessel wall (25%) can be used to treat guidewire fractures; however, another study^[11]^ reported an untreated guidewire fracture that did not result in any specific complications during 2 years of follow-up.

**Figure 2.**
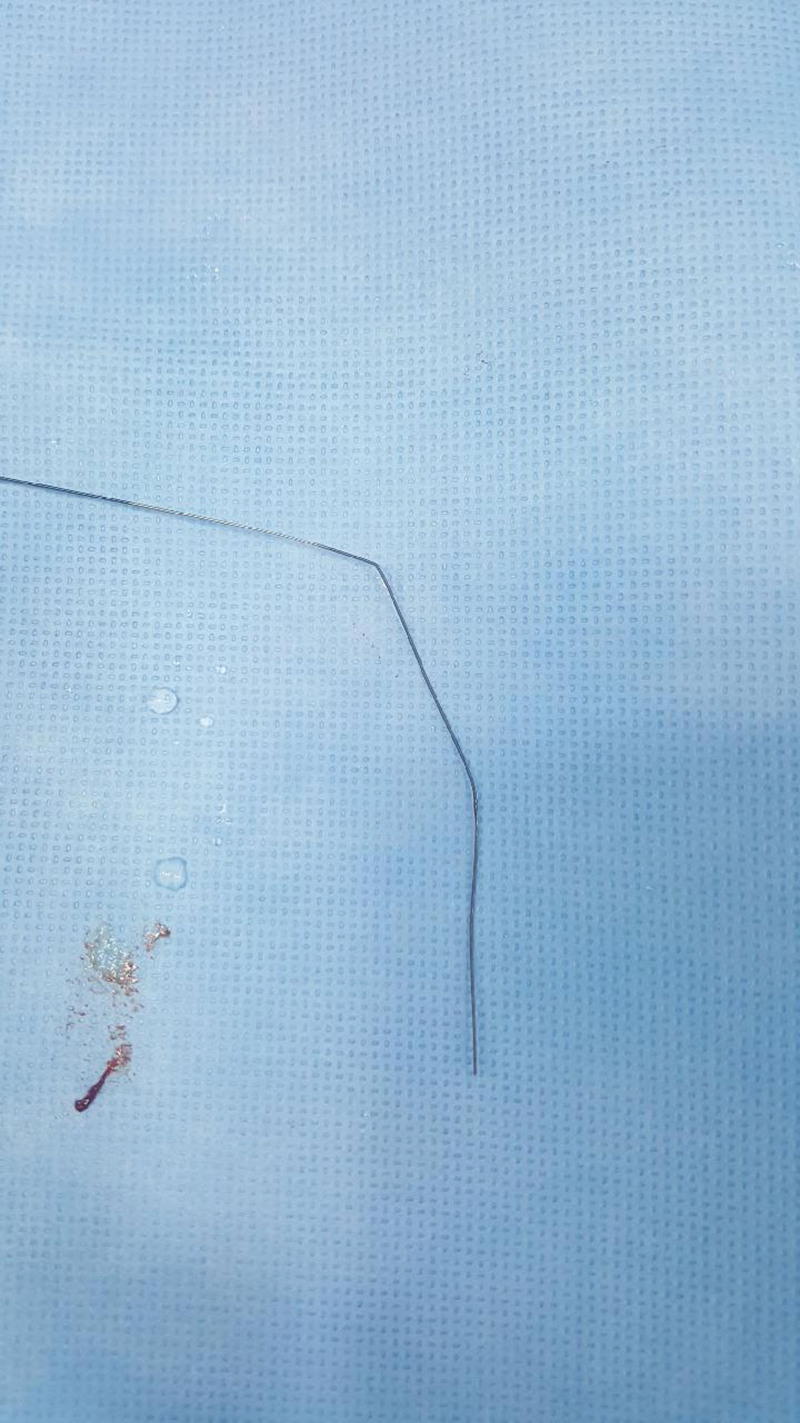
Intraprocedural image of another patient. The distal portion of the guidewire was bent during percutaneous nephrostomy.

To the best of our knowledge, there have been no reported cases of guidewire fractures during procedures involving the urinary tract. We suspect that this is because there is no resistance against the guidewire during most PCN procedures performed to resolve hydronephrosis. However, during PCN for PNL, resistance may occur when the guidewire is advanced to a space that is very narrow because the stone fills the inside of the pelvocalyx, which may cause the guidewire to bend. We have encountered several cases in which the borders of the proximal and distal parts of the guidewire were bent and scratched by the puncture needle (Fig. 2). However, for such cases, we did not attempt to further advance the wire; instead, we simply replaced it with another wire. Based on the CT findings of our case, we determined that the retained segment had penetrated the calyceal wall. We suspected that when we attempted to advance the guidewire toward the ureter, the needle tip may have penetrated the calyceal wall. We also suspected that as we pulled the wire to dislodge it from the renal parenchyma, the covered portion of the fractured segment may have been pulled like a spring; subsequently, the re-coil likely moved the segment outside the calyx, causing it to become lodged between the renal parenchyma and pelvis.

## 4. Conclusions

Cases involving fractured guidewires that remain in the renal parenchyma and perirenal space, as in our case, have not been reported previously. In our case, the retained guidewire segment caused inflammation and infection; however, the symptoms were improved with inpatient treatment. The patient reported no specific symptoms during 5 years of outpatient follow-up after hospital discharge. When performing PCN to remove renal stones, the possibility of a guidewire fracture must be considered. If resistance or scraping is felt while handling the guidewire, then it should be replaced. Additionally, if the guidewire cannot be pulled out, then the guidewire and needle should be removed together, and a new access path should be created.

## Author contributions

**Supervision:** Yook Kim, Jisun Lee, Kyung Sik Yi, Sang-Cheol Lee.

**Writing – original draft:** Seunghoon Oh, Bum Sang Cho.

**Writing – review & editing:** Seunghoon Oh, Bum Sang Cho.
